# Successful topical treatment of human biofilms using multiple antibiotic elution from a collagen-rich hydrogel

**DOI:** 10.1038/s41598-024-54477-z

**Published:** 2024-03-07

**Authors:** Ayushi D. Sharma, Evan H. Jarman, Krutika Kuppalli, Matthew J. Murphy, Michael T. Longaker, Geoffrey Gurtner, Paige M. Fox

**Affiliations:** 1 Division of Plastic & Reconstructive Surgery, Department of Surgery, Stanford University School of Medicine, Stanford, CA USA; 2 Division of Plastic & Reconstructive Surgery, Veterans Affairs Palo Alto Health Care System, Palo Alto, CA USA; 3 Division of Plastic & Reconstructive Surgery, Baylor Scott & White Medical Center, Temple, TX USA; 4 Department of Surgery, The University of Arizona College of Medicine, Tuscon, AZ USA

**Keywords:** Biofilms, Antibiotics

## Abstract

Chronic non-healing wounds significantly strain modern healthcare systems, affecting 1–2% of the population in developed countries with costs ranging between $28.1 and $96.8 billion annually. Additionally, it has been established that chronic wounds resulting from comorbidities, such as peripheral vascular disease and diabetes mellitus, tend to be polymicrobial in nature. Treatment of polymicrobial chronic wounds with oral and IV antibiotics can result in antimicrobial resistance, leading to more difficult-to-treat wounds. Ideally, chronic ulcers would be topically treated with antibiotic combinations tailored to the microbiome of a patient’s wound. We have previously shown that a topical collagen-rich hydrogel (cHG) can elute single antibiotics to inhibit bacterial growth in a manner that is nontoxic to mammalian cells. Here, we analyzed the microbiology of cultures taken from human patients diagnosed with diabetes mellitus suffering from chronic wounds present for more than 6 weeks. Additionally, we examined the safety of the elution of multiple antibiotics from collagen-rich hydrogel in mammalian cells in vivo. Finally, we aimed to create tailored combinations of antibiotics impregnated into cHG to successfully target and treat infections and eradicate biofilms cultured from human chronic diabetic wound tissue. We found that the majority of human chronic wounds in our study were polymicrobial in nature. The elution of multiple antibiotics from cHG was well-tolerated in mammalian cells, making it a potential topical treatment of the polymicrobial chronic wound. Finally, combinations of antibiotics tailored to each patient’s microbiome eluted from a collagen-rich hydrogel successfully treated bacterial cultures isolated from patient samples via an in vitro assay.

## Introduction

Chronic wounds significantly strain modern healthcare systems, affecting 1–2% of the population in developed countries with costs ranging between $28.1 and $96.8 billion annually ^1^. These wounds are often comorbid with other pathologies, such as vascular disease and diabetes mellitus, which further complicate healing. Polymicrobial colonization, infection, and biofilm formation create additional barriers to wound healing. Wounds can become gradually colonized or infected with microbes, which often create complex biofilms and can require multiple rounds of oral and/or intravenous (IV) antibiotic therapy. Serial and prolonged treatment courses can lead to the development of antimicrobial resistance, making infections increasingly difficult to treat. Renal and hepatic clearance of antibiotics can further limit the dosages and combinations of antibiotics that can be given due to patient comorbidities. As a result, systemic antibiotic treatment is often a suboptimal treatment strategy for chronic wound infections.

The treatment of chronic wounds is made more difficult by the presence of biofilms; it is estimated that 80% of chronic wounds are complicated by biofilm formation ^2^. Furthermore, microbial compositions can vary over time within tissues and antibiotic administration is known to alter the microbiome of chronic wounds ^3^. Similarly, the presence or absence of certain co-morbid conditions, such as diabetes and cancer, can dramatically alter the microbial composition of chronic wounds ^4–6^. Diabetic chronic wounds, specifically, have mycobiomes and bacteriomes that change in response to the microenvironment of the wound ^5^.

The ideal way to treat chronic wounds is topically with personalized antimicrobial combinations tailored to the microbiome of each patient’s wound. Personalized topical treatment would eliminate the side effects of systemically administered antibiotics while targeting specific microbes present in the wound thereby eradicating biofilms. Localized release and delivery models are ideal to elute antibiotics steadily and safely.

We conducted a literature review to analyze the most common bacteria isolated from chronic wounds. Twelve articles were selected for review after applying relevant inclusion criteria (systematic reviews, randomized controlled trials, and outcome studies) and excluding animal studies, case reports, and literature not written in English. The most common bacteria identified in chronic wounds are Methicillin -resistant, and Methicillin-sensitive *Staphylococcus aureus (MRSA and MSSA)*, *Pseudomonas aeruginosa, Enterococcus* spp., and beta-hemolytic *streptococci*. These bacteria are widely considered the primary causes of infection and nonhealing in chronic wounds ^7–10^. Other common microorganisms isolated from chronic wounds include *Enterobacter* spp., *Bacteroides* spp., *Peptostreptococcus* spp., and *Fusobacterium* spp. ^11^. Of note, data are limited by the use of traditional microbial isolation techniques that self-select for more fastidious organisms ^11,12^.

The goal of this study was three-fold. First to demonstrate the safe elution of multiple antibiotics from a collagen-rich hydrogel, second to define the changing microbiome of human chronic wounds over time and lastly to demonstrate in vitro that these unique bacterial signatures could be effectively treated with hydrogel augmented with antibiotics customized to the patient. We have previously shown that a topical collagen-rich hydrogel (cHG) can provide controlled elution of multiple, individually delivered antibiotics leading to inhibition of bacterial growth and biofilm elimination while avoiding cytotoxicity of host cells in a murine stented wound model ^13^. This work builds on our prior work by translating cHG + antibiotics (cHG + abx) to human biofilms.

## Methods

The study was approved by the Stanford Institutional Review Board and all experiments were performed in accordance with relevant guidelines and regulations.

### Collagen hydrogel formation

We synthesized 2.5% collagen-rich thermoresponsive hydrogel (cHG) according to previously established protocols ^13^. Briefly, flexor tendons were harvested from adult human cadavers (Science Care, Phoenix, Arizona) and decellularized, lyophilized, and made into a powder. Samples were digested with 1 mg/mL of pepsin (Sigma-Aldrich, St. Louis, MO) at a pH of about 2.2–2.4 for 14 h. The reaction was then neutralized to a pH of 7.4, and the hydrogel was examined microscopically for quality. Gelation was then confirmed after incubation for one hour at 37 °C.

### cHG-antibiotic composite preparation

Gentamicin sulfate (Thermo Fischer Scientific, Waltham, MA), ciprofloxacin (Thermo Fischer Scientific), imipenem monohydrate (Thermo Fischer Scientific), clindamycin hydrochloride monohydrate (Thermo Fischer Scientific), trimethoprim (Thermo Fischer Scientific), and sulfamethoxazole (Thermo Fischer Scientific) were prepared to form stock solutions according to manufacturer’s instructions. These antibiotics were selected based on clinical applicability and antibiogram data via Stanford Health Care. Stock antibiotic solutions were mixed with liquid cHG, and 10 μL of the solution was placed onto a polycarbonate filter membrane.

Based on the most common bacterial species identified in the literature and their resistance profiles, we tested the toxicity of the following six antibiotic combinations: Clindamycin + Ciprofloxacin, Ciprofloxacin + Imipenem, Clindamycin + Gentamicin, Clindamycin + Imipenem, Gentamicin + Ciprofloxacin, and trimethoprim/sulfamethoxazole (TMP/SMX) + Gentamicin. Vancomycin was excluded due to its high molecular weight and lack of integration into cHG. For each antibiotic, we used antibiotic concentrations within or at 100× the minimum inhibitory concentration (MIC) by the Clinical and Laboratory Standards Institute (CLSI). A high MIC was selected since antibiotic resistance within a biofilm can reach 10–10,000× the normal bacterial MIC. Final concentrations are shown in Table 1^14^.

**Table 1:** Antibiotic concentrations.

Antibiotic	Concentration	MIC	Bacteria used to calculate MIC
Ciprofloxacin	200 μg/mL	100× upper limit	*Enterococcus faecalis *
Imipenem	400 μg/mL	100× upper limit	*Pseudomonas aeruginosa *
TMP/SMX	3.2+60.8 mg/mL	100× upper limit	*Pseudomonas aeruginosa *
Gentamicin	1 mg/mL	100× MIC range	*Enterococcus faecalis *
Clindamycin	100 μg/mL	100× lower limit	*Enterococcus faecalis *

Concentrations used for each antibiotic in relation to each antibiotic’s MIC.

MIC, minimum inhibitory concentration; TMP/SMX, trimethoprim-sulfamethoxazole; MRSA, methicillin-resistant *Staphylococcus aureus*.

The hydrogel and antibiotic (cHG + abx) composites were pipetted onto polycarbonate membranes and incubated for 1 h at 37 °C to confirm gelation. Based on the speciation for each chronic wound, four possible antibiotic combinations were selected to obtain the best antimicrobial coverage based on the Stanford Healthcare Antibiogram ^15^ and integrated within the cHG.

### Cytotoxicity in mammalian cells

Mouse and human adipose derived stem cells (ASC) and fibroblasts (FB) (Cell Applications, San Diego, CA) were incubated at 37 °C in a 5% CO^2^ humidifier and grown to 95% confluence in cell-specific media. We chose these cell lines to demonstrate safety of the antibiotic eluting cHG (cHG + abx) in differentiated and undifferentiated mammalian cells in an animal model (mouse) and a human model. Cells were seeded onto 24-well culture plates at a density of 2 × 10^4^ cells/mL and were incubated for 24 h to allow for cell attachment. cHG + abx treatments were added to the wells and incubated until set time points (12 h, 24 h, 48 h, and 72 h). We then performed a LIVE/DEAD viability/cytotoxicity assay (Thermo Fischer Scientific) as per manufacturer’s instructions. In brief, cell media supernatant was removed, and the LIVE/DEAD reagent was pipetted into to the wells. The cells were then incubated at 27 °C for 30 min. Cells were visualized using a KEYENCE fluorescence microscope (BZ-X700; KEYENCE, Osaka, Japan). The images were analyzed using MATLAB (Mathworks, Natick, MA). All experiments were conducted in triplicate.

### Patient chronic wound microbiomes

After Institutional Review Board (IRB-62832) approval, patient samples were collected monthly for three months from the Stanford Advanced Wound Care Center (AWCC) in Redwood City, CA. Ethics approval was obtained and informed consent given by each patient participating in this study. Wound slough was obtained via curettage during standard wound debridement. One half of each sample was placed in a 1:5 glycerol stock, and frozen at − 80 °C immediately. The second half of the sample was sent to the Stanford Healthcare clinical microbiology lab for independent identification of bacterial species.

### Bacterial growth conditions

Wound samples were removed from − 80 °C and divided into two tubes, one to be grown in anoxic conditions and another in oxygen-rich conditions, both grown in tryptic soy broth media shaking at 37 °C for 14 h. Bacterial cultures were used at mid-log growth phase, between 0.2 and 0.3 by absorbance at 600 nm.

### Bacterial strain reverse transcriptase-PCR identification

Composition of the laboratory grown wound slough was confirmed using rt-PCR. Bacteria were isolated and amplified either using target gene primers or 16sRNA. rt-PCR results were compared to clinical lab results for confirmation of wound biodiversity.

### Modified Kirby-Bauer assay

A modified Kirby-Bauer disk diffusion assay was utilized to study the inhibition of bacterial growth by cHG + abx composites. A 200 μL aliquot of bacteria at 10^6^ CFU/mL was plated on MacConkey Blood agar plates. For anaerobes, sealed culture bags with oxygen sinks were used to promote bacterial growth. Plates were then incubated at 37 °C for 1 h. cHG + abx composites were pre-eluted in phosphate buffered saline (PBS) and then placed on the agar plates. Pre-elution is used to determine how much effective antibiotic remains within the gel at set timepoints. The plates were then incubated at 37 °C for 24 h. The diameter of the zone of inhibition (ZOI) was measured. The average of two measurements was used per experiment, and each condition was performed in triplicate. Images were also obtained.

### Fluorometric biofilm disruption

A polycarbonate membrane was placed on a MacConkey Blood agar plate, and 50 μL of bacterial suspension adjusted to 10^6^ CFU/mL was pipetted onto the membrane and distributed evenly with a sterile cotton tip. Membranes were then incubated anaerobically and/or aerobically, depending on the culture results of the sample, for 14 h at 37 °C to create a biofilm. After incubation, the biofilm was removed and placed in a well. Biofilms were incubated for 24 h at 37 °C in either untreated or treated with cHG + abx composite used immediately (0 h) or pre-eluted for 24 h. The supernatant was poured off, and a 1 mL aliquot of the nucleic acid stain SYTO61 (Invitrogen, Carlsbad, CA) was diluted 1:100 in PBS and added to each well. Plates were incubated for 30 min at room temperature and then rinsed with fresh PBS. The membrane biofilms were imaged with a KEYENCE fluorescence microscope (BZ-X700; KEYENCE, Osaka, Japan) and analyzed using ImageJ. Integrated density values were obtained ^15^.

### Statistical analysis

Statistical analyses were conducted using PRISM software (GraphPad Software, San Diego, CA) and results are reported in units + /− standard deviation. Differences were evaluated either relative to control using an unpaired *t* test, or relative to other time points using ANOVA. Significance was set to a *p* value of < 0.05.

## Results

### Cytotoxicity in mammalian cells

**Figure 1:**
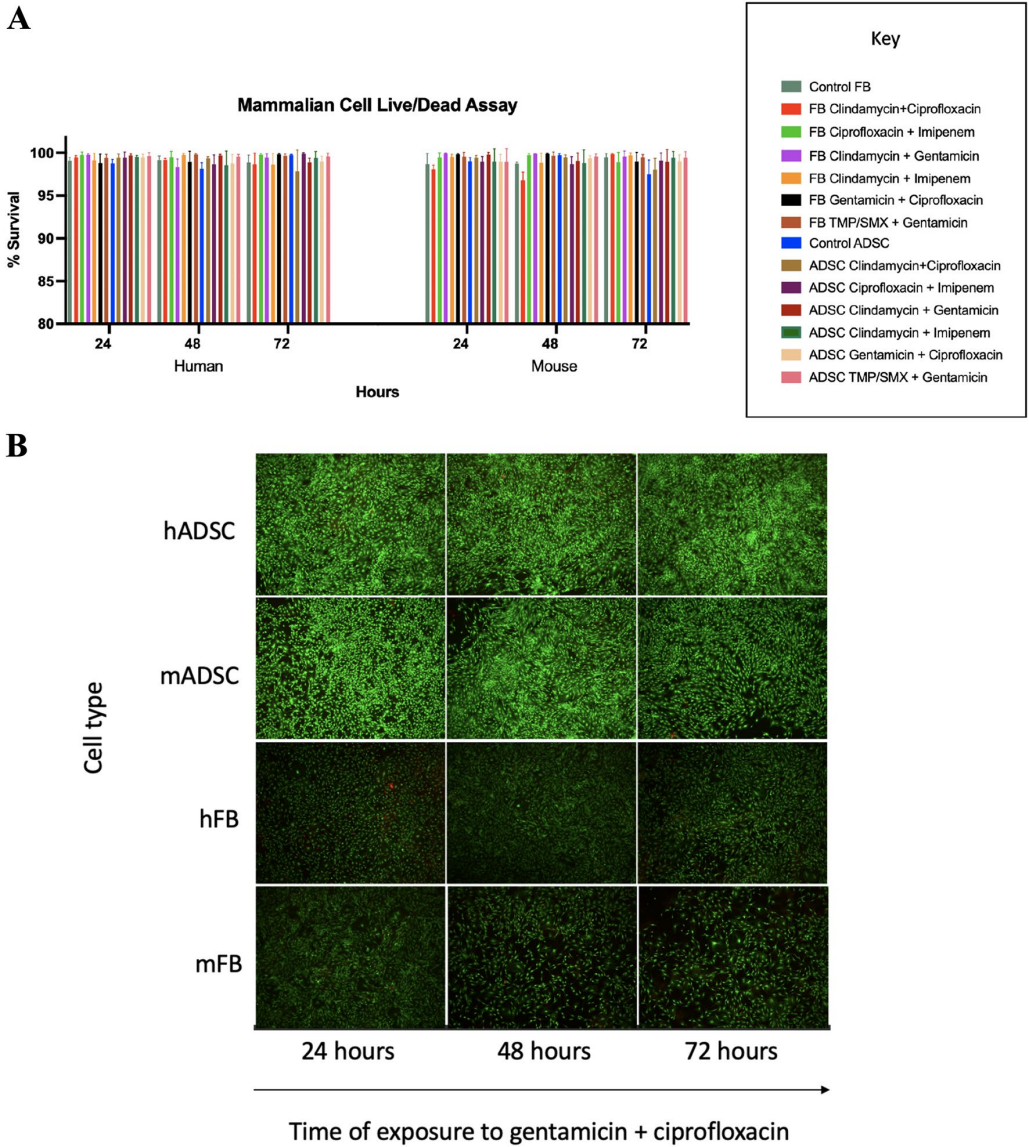
Cytotoxicity in mammalian cells. (**A**) The cytotoxicity of collagen-rich hydrogel with antibiotic(s) (cHG + abx) composite on adipose derived stem cells (ADSC) and fibroblasts (FB) in human and mouse cell lines was assessed with a LIVE/DEAD assay. The percentage of viable cells are shown over 72 h. (**B**) Representative images of LIVE/DEAD staining of gentamicin and ciprofloxacin in cHG. Green represents living, viable cells and red represents dead, nonviable cells. hADSC, human adipose derived stem cells; mADSC, mouse adipose derived stem cells; hFB, human fibroblasts; mFB, mouse fibroblasts.

When human and mouse fibroblasts (FBs) and adipose derived stem cells (ADSCs) were exposed to cHG + abx over 72 h, no significant difference in cell survival was seen in treatments group compared to control (Fig. 1A). Cellular densities also remained relatively stable over time among both the treatment and control groups (Fig. 1B).

### Patient chronic wound microbiomes

**Figure 2:**
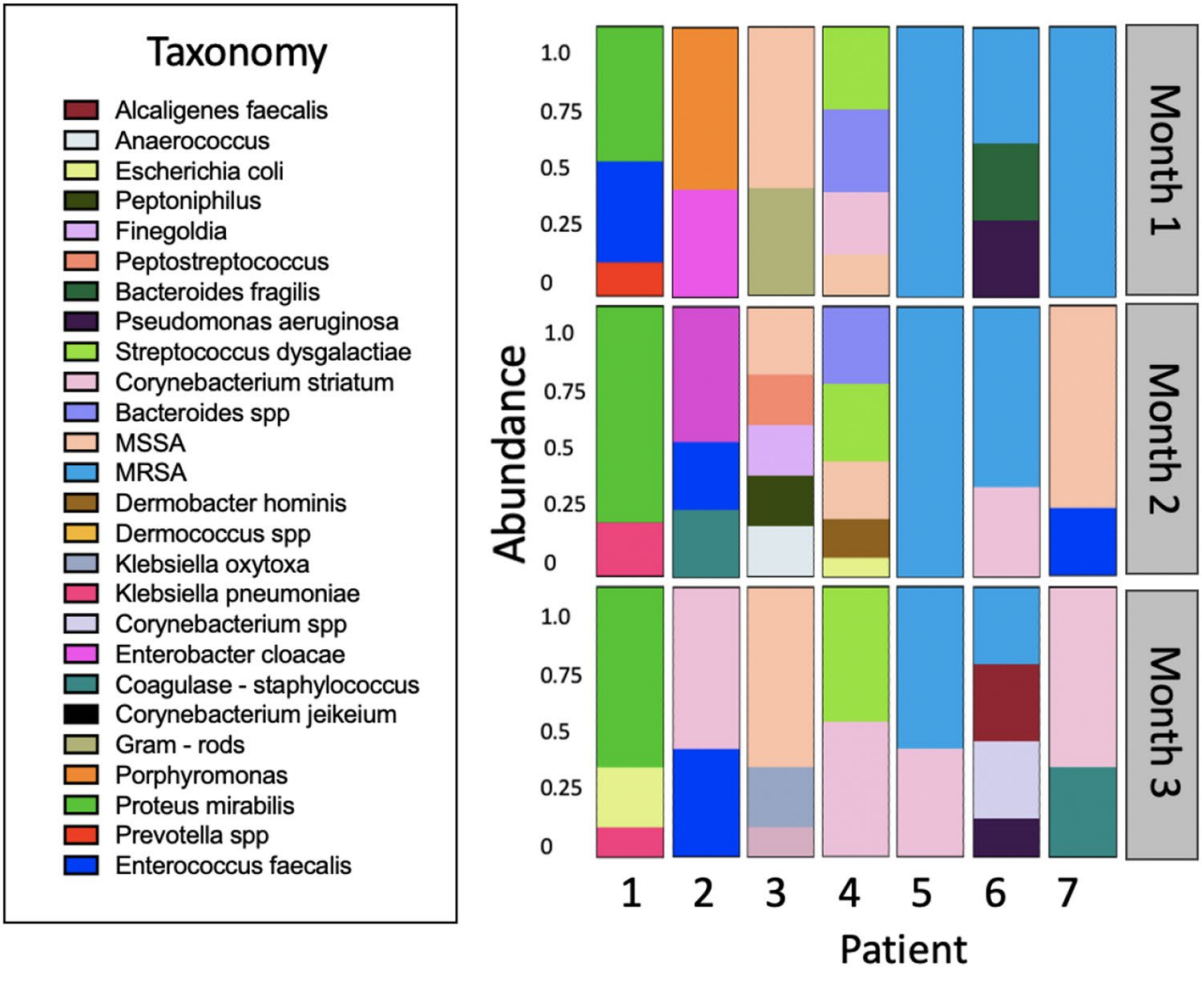
Bacterial composition of chronic diabetic wounds sampled from seven patients over a period of 3 months. Abundance is listed as a relative density of the total wound composition as determined by the Stanford Clinical Microbiology Lab.

Speciation of the microbiome of 21 chronic diabetic wound samples from 7 patients over 3 months is shown in Fig. 2.

### Modified Kirby-Bauer assay

**Figure 3:**
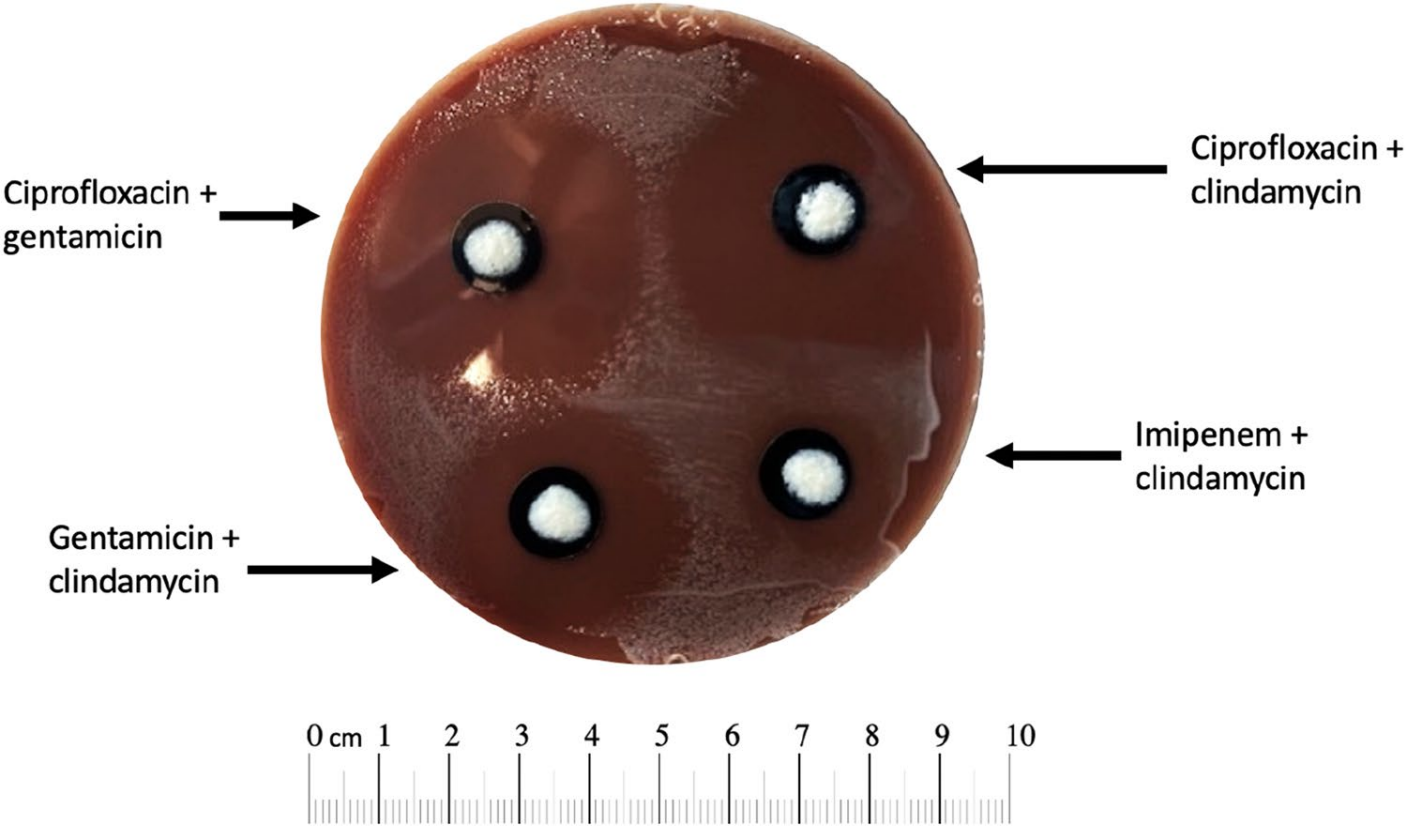
Sensitivity Testing of Patient 2, Month 2, aerobes. Sensitivity testing to select optimal antibiotic combination to target bacterial composition of the wound.

Antibiotic combinations were tested against bacterial lawns formed by cultured wound slough over a period of 24 h (Fig. 3) to determine the most effective antibiotic or combination of antibiotics for bacterial elimination. The antibiotic regimen with the largest zone of inhibition (ZOI) was selected for each patient sample for further testing (Table 2).

**Table 2:** Sensitivity Testing Results.

Patient	Antibiotic(s) used		
	Month 1	Month 2	Month 3
1	Ciprofloxacin + clindamycin	Clindamycin + gentamicin	Ciprofloxacin + imipenem
2	Clindamycin + gentamicin	Ciprofloxacin + gentamicin	Gentamicin + imipenem
3	Clindamycin + imipenem	Clindamycin + imipenem	Gentamicin + imipenem
4	Ciprofloxacin + clindamycin	Clindamycin + imipenem	Clindamycin + gentamicin
5	Clindamycin	Clindamycin	Clindamycin + gentamicin
6	Clindamycin + gentamicin	Clindamycin + gentamicin	Clindamycin + gentamicin
7	Gentamicin	Clindamycin + gentamicin	Clindamycin + gentamicin

The optimal antibiotic(s), with the greatest zone of inhibition (ZOI) were selected for the wounds of seven patients over 3 months.

**Figure 4:**
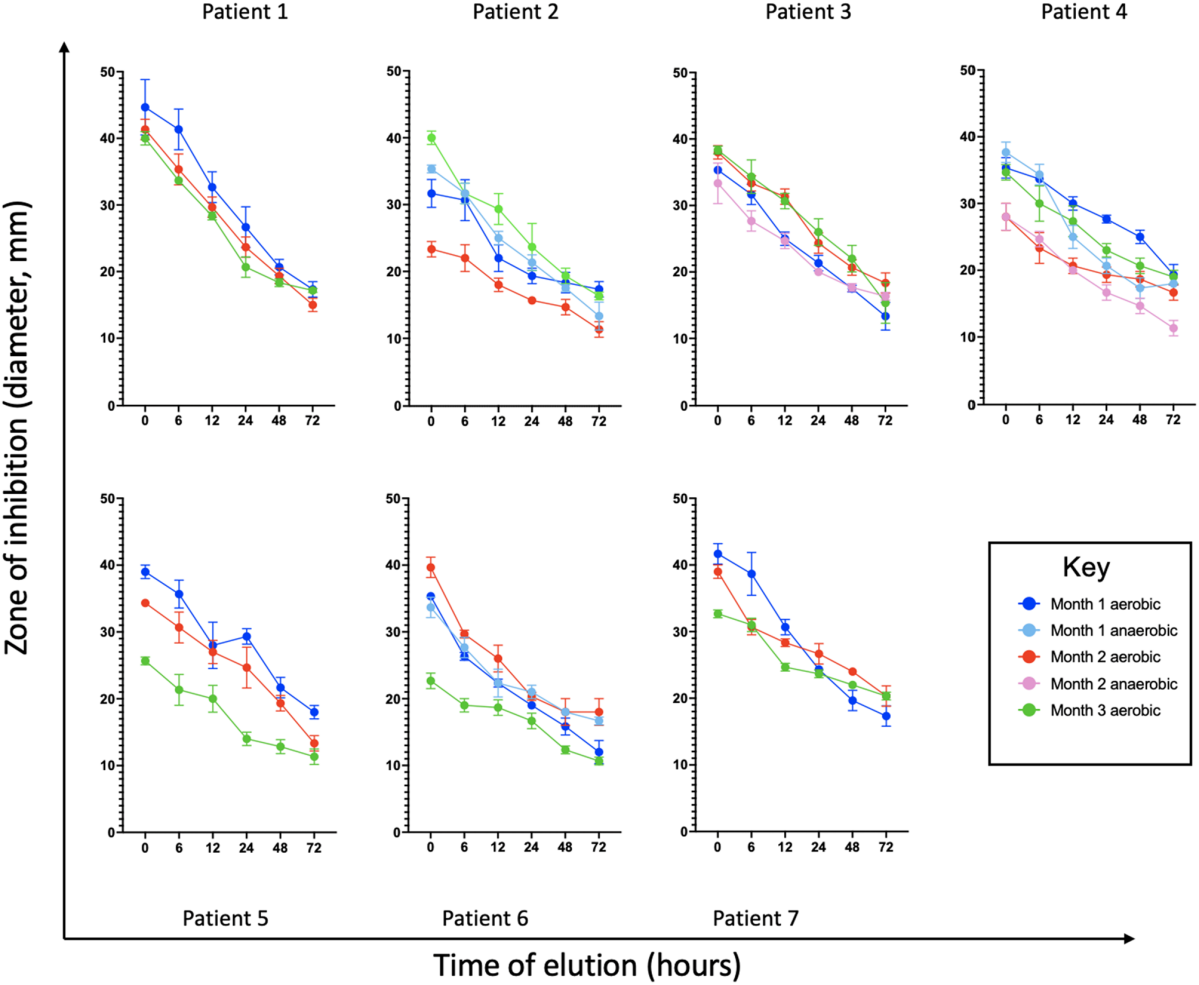
Kirby Bauer Assay. Zone of inhibition over a period of 72 h in bacteria isolated from patient diabetic wounds over a period of three months and grown in vitro treated with their optimal antibacterial therapies.

Figure 4 shows the modified Kirby-Bauer assay over time for each patient slough. The ZOI, representing bacterial inhibition, was highest in the cHG + abx composite used immediately, while the ZOI decreased when the composite was pre-eluted to 48 and 72 h. In general, the amount of bacterial inhibition decreased as the pre-elution duration increased as expected. Significance values between time points in each patient sample can be found in Appendix A.

### Fluorometric biofilm disruption

**Figure 5:**
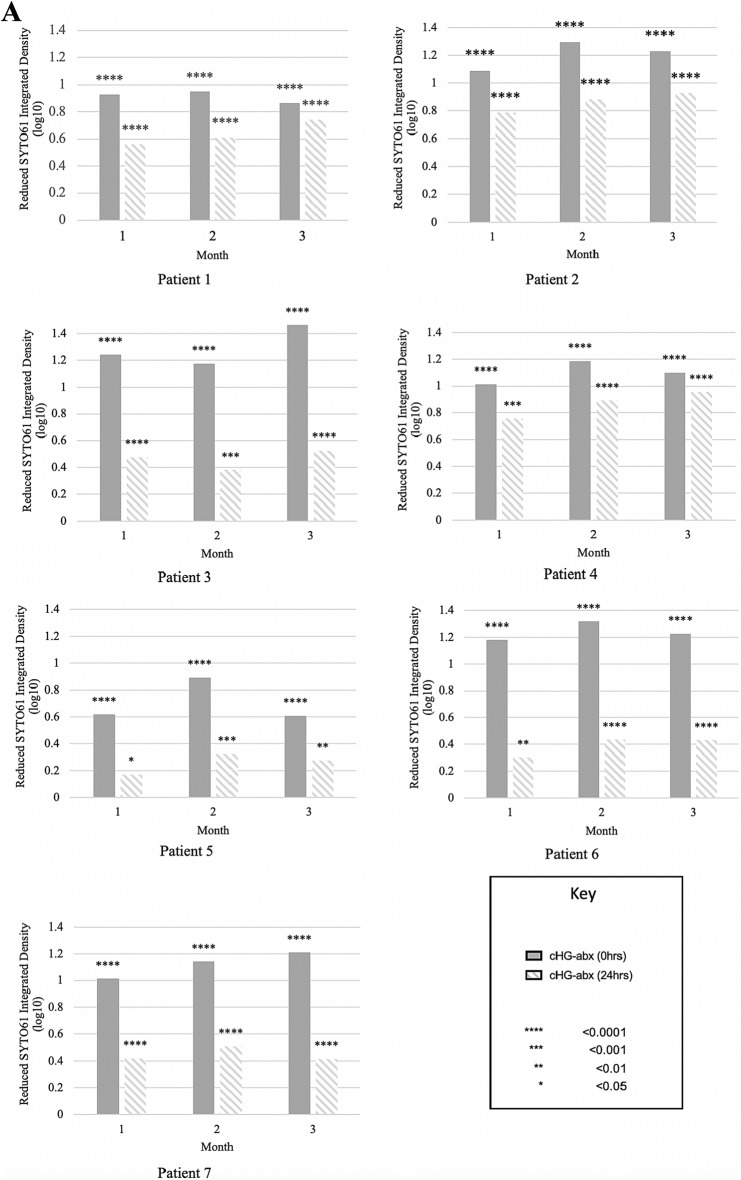

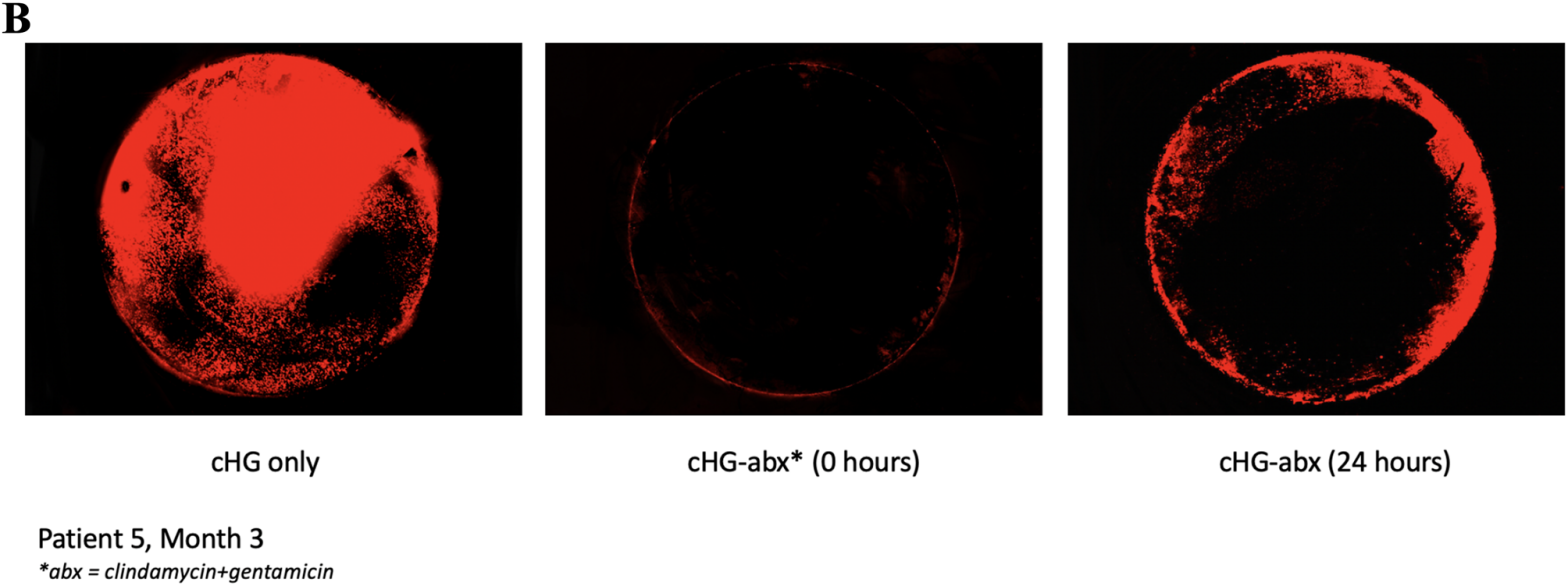
SYTO61 Biofilm eradication. (**A**) Hydrogels were either cHG only (control), loaded with optimal, wound-specific antibiotics not pre-eluted (0 h) or pre-eluted to 24 h, and then used to treat biofilms formed by chronic wounds. (**B**) Patient five, month three chronic wound biofilm either treated with blank hydrogel (cHG only), tailored antibiotics (clindamycin and gentamicin) not pre-eluted (0 h), or antibiotics pre-eluted for 24 h. All combinations were statistically effective at biofilm elimination 0 and 24 h of pre-elution. Red fluorescence indicates the presence of biofilm.

There was significant biofilm disruption at both 0 h pre-elution and 24 h pre-elution as compared to cHG only control in all patients’ chronic wound biofilms (Fig. 5A). At 24 h pre-elution, residual biofilm remained only at the periphery of the biofilm, and at 0 h pre-elution there was qualitatively very little biofilm remaining. A sample biofilm disruption assay result is shown in Fig. 5B.

## Discussion

Collagen-rich hydrogel augmented with targeted antibiotics is a promising avenue for the topical treatment of chronic diabetic wounds. Patient chronic wound samples in this study were primarily polymicrobial in nature, and their compositions changed over time. Characterization of a colonized or infected wound is an important step in the customization of topical antibiotic treatments. Currently, topical chronic wound care treatments do not target specific bacteria growing in wounds. Bacteria and complex biofilms are likely less susceptible to traditional antibiotic therapies due to rising rates of antimicrobial resistance, and some therapies carry additional risks if used for prolonged periods.

This study demonstrates three key features of cHG for chronic wound treatment: (1) the safety of cHG-abx in mammalian cells, (2) the utility of cHG as a vector for antibiotic delivery, and (3) its efficacy in reducing bacterial burden and treating biofilm formation in cultures isolated from human wounds. This is an important advancement in the potential for personalization of chronic wound treatment based specifically on bacterial speciation and sensitivity profiles.

The microbiome of chronic diabetic wounds is complex, and treatment of colonized and infected chronic wounds remains poor. Several studies in recent years have explored the dynamicity of the microbiome of chronic wounds and the efficacy of potential treatment options. Verbanic et al. explored the effect of sharp debridement, a widely used mechanical treatment for chronic wounds, on the microbial composition of wounds. The composition did not change before and after treatment in this study, but chronic wounds present for longer than six months had more aerobes and facultative anaerobes than at the start of the study ^16^. Similarly, a study by Drago et al. ^17^ looked at sharp mechanical debridement as a primary treatment method for complex, polymicrobial chronic wounds, with no mention of a topical or systemic treatment. There is a paucity of studies examining the complexity of the chronic wound microbiome and the effect of targeted topical or systemic antimicrobial treatments. Studies specifically examining topical treatment of chronic diabetic wounds use time to closure, wound healing rate, amputation and wound recurrence as outcomes of interest making it unclear what each treatment’s effects are on biofilm eradication and infection control ^18–22^.

Our collagen-rich hydrogel has the potential to not only accelerate healing of chronic wounds, but eradicate infection and biofilms ^13^. Biofilm formation is a significant barrier to the treatment of chronic wounds making elimination of the biofilm critical to wound healing. Hydrogels have inherent wound healing properties making them an attractive carrier of antibiotics ^23^. Additionally, there are known advantages to using biocompatible materials such as the human-derived collagen used in this study. Biocompatible materials such as collagen produce less immunologic and inflammatory reaction, while providing biomaterials that accelerate and improve wound healing ^24–26^. Collagen-rich hydrogels contain fibrinogen and thrombin which are key factors in wound healing, and provide a tissue-like environment that promotes cellular regeneration ^27^. While hydrogel alone has known wound-healing properties, the opportunity to augment and tailor hydrogels cannot be understated. Collagen covalent cross linkages are widely used in drug delivery and tissue engineering due to their high tensile strength, flexibility, and hydrophilic properties ^28,29^.

Depending on the characteristics of the chronic wound, different drug regimens can be considered. Analyzing antibiograms of the most common microbes isolated from colonized chronic diabetic wounds suggest that a combination of drugs would be the best approach to adequately cover for all species colonizing a chronic wound. While there are multiple antibiotic permutations, the most common clinical multi-drug regimens for moderate to severe polymicrobial diabetic wounds include a second or third generation cephalosporin plus clindamycin and a fluoroquinolone plus vancomycin ^30–32^. Additionally, there are studies that demonstrate that the addition of a carbapenem has a synergistic effect on the anti-MRSA properties of vancomycin therapy ^31,33^. However, the systemic delivery of these antibiotics produces a wide variety of side effects; therefore, a topical delivery method would represent a significant advance in wound care. Additionally, cHG + abx could be considered as an adjunct treatment to systemic antibiotic by augmenting the gel with less commonly used or poorly tolerated systemic antibiotics that would remain effective topically.

Our study showed steady release of antibiotic over a period of up to 72 h, as demonstrated by the amount of bacterial inhibition seen in cHG + abx treated bacterial cultures harvested from patient chronic wounds. While we have previously shown that this cHG is biocompatible and safely tolerated, augmenting the hydrogels with several combinations of antibiotics did not change its safety profile ^8^.

Clinically, all patients were treated with serial debridement with curettage. One patient, Patient 4, was started on oral antibiotics during our study. While this did not directly affect our in vitro studies, it is important to note that changing microbiome of a wound over time can be artificially selected due to oral or IV therapies. Verbanic et al. found if chronic wounds were present for more than six months, the distribution trended towards an overabundance of facultative anaerobic organisms without mention of systemic therapy. The composition of the wound in Patient 4 started off polymicrobial and was ultimately entirely comprised of *Streptococcus dysgalactiae* and *Corynebacterium striatum*, both facultative anaerobes. In fact, facultative anaerobes made up the largest ratio of microbes isolated in all three-month samples in this study.

The major limitation of this study is the use of an in vitro model. Biofilms are complex ecosystems that are often produced by several bacteria, viruses and fungi that synergistically increase the pathogenicity of the biofilm ^34–36^. This complexity is not mimicked in vitro. Additionally, we only examined bacteria within each sample. To mitigate these limitations, we used a clinical lab and rt-PCR to verify that we were able to reproduce the bacterial composition for each sample. Future work will utilize direct sample analysis to identify difficult to culture bacteria, virus, and fungi within patient samples. It is also important to note that while efforts were made to eradicate any infection present in cultures that were grown in this study, not all bacterial isolates would necessarily be treated in a clinical setting.

This study did not explore the direct kinetics of antibiotic release from cHG. The complex dynamic release of multiple antibiotic combinations is under investigation. The indirect methods used here demonstrated in vitro cultured bacteria were effectively treated with cHG-abx pre-eluted up to 72 h; however, the exact rate and trajectory of multiple antibiotic release is not yet well understood.

Human-derived collagen-rich hydrogel is an effective vector for safe delivery of topical antibiotics to treat bacteria and biofilms from human chronic diabetic wound slough.

### Supplementary Information

Supplementary Information 1.

Supplementary Information 2.

## Data Availability

The datasets generated and analyzed during the current study are available and attached to this submission.
