# Supplementary material for: Successful topical treatment of human biofilms using multiple antibiotic elution from a collagen-rich hydrogel
Source: Sci Rep. 2024 Mar 7;14:5621. doi: 10.1038/s41598-024-54477-z (PMC10920629; doi:10.1038/s41598-024-54477-z)
Supplement: Supplementary file 1 — Supplementary Information 1. [file 41598_2024_54477_MOESM1_ESM.docx]

| Patient 1 | | | | | |
| --- | --- | --- | --- | --- | --- |
| Month 1 aerobic | P Value | Summary | Month 2 aerobic | P Value | Summary |
| 0 vs. 6 | 0.40 | ns | 0 vs. 6 | 0.24 | ns |
| 0 vs. 12 | 0.03 | * | 0 vs. 12 | 0.02 | * |
| 0 vs. 24 | 0.16 | ns | 0 vs. 24 | 0.02 | * |
| 0 vs. 48 | 0.06 | ns | 0 vs. 48 | 0.01 | ** |
| 0 vs. 72 | 0.04 | * | 0 vs. 72 | 0.001 | *** |
| 6 vs. 12 | 0.02 | * | 6 vs. 12 | 0.08 | ns |
| 6 vs. 24 | 0.18 | ns | 6 vs. 24 | 0.11 | ns |
| 6 vs. 48 | 0.05 | * | 6 vs. 48 | 0.05 | ns |
| 6 vs. 72 | 0.02 | * | 6 vs. 72 | 0.03 | * |
| 12 vs. 24 | 0.54 | ns | 12 vs. 24 | 0.24 | ns |
| 12 vs. 48 | 0.09 | ns | 12 vs. 48 | 0.07 | ns |
| 12 vs. 72 | 0.05 | * | 12 vs. 72 | 0.02 | * |
| 24 vs. 48 | 0.12 | ns | 24 vs. 48 | 0.02 | * |
| 24 vs. 72 | 0.12 | ns | 24 vs. 72 | 0.02 | * |
| 48 vs. 72 | 0.13 | ns | 48 vs. 72 | 0.02 | * |
| Month 3 aerobic | P Value | Summary |  |  |  |
| 0 vs. 6 | 0.01 | * |  |  |  |
| 0 vs. 12 | 0.01 | * |  |  |  |
| 0 vs. 24 | 0.01 | * |  |  |  |
| 0 vs. 48 | 0.01 | ** |  |  |  |
| 0 vs. 72 | <0.0001 | **** |  |  |  |
| 6 vs. 12 | 0.01 | * |  |  |  |
| 6 vs. 24 | 0.03 | * |  |  |  |
| 6 vs. 48 | 0.01 | ** |  |  |  |
| 6 vs. 72 | <0.0001 | **** |  |  |  |
| 12 vs. 24 | 0.08 | ns |  |  |  |
| 12 vs. 48 | 0.01 | * |  |  |  |
| 12 vs. 72 | 0.01 | ** |  |  |  |
| 24 vs. 48 | 0.24 | ns |  |  |  |
| 24 vs. 72 | 0.19 | ns |  |  |  |
| 48 vs. 72 | 0.37 | ns |  |  |  |

| Patient 2 | | | | | |
| --- | --- | --- | --- | --- | --- |
| Month 1 aerobic | P Value | Summary | Month 1 anaerobic | P Value | Summary |
| 0 vs. 6 | 0.99 | ns | 0 vs. 6 | 0.16 | ns |
| 0 vs. 12 | 0.03 | * | 0 vs. 12 | 0.002 | ** |
| 0 vs. 24 | 0.01 | * | 0 vs. 24 | 0.002 | ** |
| 0 vs. 48 | 0.01 | ** | 0 vs. 48 | <0.0001 | **** |
| 0 vs. 72 | 0.01 | ** | 0 vs. 72 | 0.01 | ** |
| 6 vs. 12 | 0.10 | ns | 6 vs. 12 | 0.03 | * |
| 6 vs. 24 | 0.06 | ns | 6 vs. 24 | 0.01 | ** |
| 6 vs. 48 | 0.04 | * | 6 vs. 48 | 0.01 | ** |
| 6 vs. 72 | 0.04 | * | 6 vs. 72 | 0.002 | ** |
| 12 vs. 24 | 0.48 | ns | 12 vs. 24 | 0.08 | ns |
| 12 vs. 48 | 0.30 | ns | 12 vs. 48 | 0.01 | ** |
| 12 vs. 72 | 0.16 | ns | 12 vs. 72 | 0.02 | * |
| 24 vs. 48 | 0.93 | ns | 24 vs. 48 | 0.07 | ns |
| 24 vs. 72 | 0.42 | ns | 24 vs. 72 | 0.04 | * |
| 48 vs. 72 | 0.93 | ns | 48 vs. 72 | 0.23 | ns |
| Month 2 aerobic | P Value | Summary | Month 3 aerobic | P Value | Summary |
| 0 vs. 6 | 0.89 | ns | 0 vs. 6 | 0.01 | * |
| 0 vs. 12 | 0.02 | * | 0 vs. 12 | 0.03 | * |
| 0 vs. 24 | 0.01 | * | 0 vs. 24 | 0.04 | * |
| 0 vs. 48 | 0.005 | ** | 0 vs. 48 | 0.0002 | *** |
| 0 vs. 72 | 0.001 | ** | 0 vs. 72 | 0.0001 | *** |
| 6 vs. 12 | 0.23 | ns | 6 vs. 12 | 0.70 | ns |
| 6 vs. 24 | 0.09 | ns | 6 vs. 24 | 0.17 | ns |
| 6 vs. 48 | 0.05 | * | 6 vs. 48 | 0.003 | ** |
| 6 vs. 72 | 0.02 | * | 6 vs. 72 | 0.01 | ** |
| 12 vs. 24 | 0.16 | ns | 12 vs. 24 | 0.37 | ns |
| 12 vs. 48 | 0.10 | ns | 12 vs. 48 | 0.03 | * |
| 12 vs. 72 | 0.01 | * | 12 vs. 72 | 0.03 | * |
| 24 vs. 48 | 0.76 | ns | 24 vs. 48 | 0.50 | ns |
| 24 vs. 72 | 0.05 | * | 24 vs. 72 | 0.22 | ns |
| 48 vs. 72 | 0.12 | ns | 48 vs. 72 | 0.12 | ns |

| Patient 3 | | | | | |
| --- | --- | --- | --- | --- | --- |
| Month 1 aerobic | P Value | Summary | Month 2 aerobic | P Value | Summary |
| 0 vs. 6 | 0.16 | ns | 0 vs. 6 | 0.04 | * |
| 0 vs. 12 | 0.002 | ** | 0 vs. 12 | 0.01 | * |
| 0 vs. 24 | 0.002 | ** | 0 vs. 24 | 0.003 | ** |
| 0 vs. 48 | <0.0001 | **** | 0 vs. 48 | 0.0003 | *** |
| 0 vs. 72 | 0.01 | ** | 0 vs. 72 | 0.001 | *** |
| 6 vs. 12 | 0.03 | * | 6 vs. 12 | 0.42 | ns |
| 6 vs. 24 | 0.01 | ** | 6 vs. 24 | 0.01 | ** |
| 6 vs. 48 | 0.01 | ** | 6 vs. 48 | 0.001 | ** |
| 6 vs. 72 | 0.002 | ** | 6 vs. 72 | 0.002 | ** |
| 12 vs. 24 | 0.08 | ns | 12 vs. 24 | 0.02 | * |
| 12 vs. 48 | 0.01 | ** | 12 vs. 48 | 0.002 | ** |
| 12 vs. 72 | 0.02 | * | 12 vs. 72 | 0.003 | ** |
| 24 vs. 48 | 0.07 | ns | 24 vs. 48 | 0.16 | ns |
| 24 vs. 72 | 0.04 | * | 24 vs. 72 | 0.05 | * |
| 48 vs. 72 | 0.23 | ns | 48 vs. 72 | 0.43 | ns |
| Month 3 aerobic | P Value | Summary |  |  |  |
| 0 vs. 6 | 0.35 | ns |  |  |  |
| 0 vs. 12 | 0.01 | * |  |  |  |
| 0 vs. 24 | 0.02 | * |  |  |  |
| 0 vs. 48 | 0.01 | * |  |  |  |
| 0 vs. 72 | 0.02 | * |  |  |  |
| 6 vs. 12 | 0.40 | ns |  |  |  |
| 6 vs. 24 | 0.06 | ns |  |  |  |
| 6 vs. 48 | 0.02 | * |  |  |  |
| 6 vs. 72 | 0.01 | ** |  |  |  |
| 12 vs. 24 | 0.16 | ns |  |  |  |
| 12 vs. 48 | 0.03 | * |  |  |  |
| 12 vs. 72 | 0.03 | * |  |  |  |
| 24 vs. 48 | 0.32 | ns |  |  |  |
| 24 vs. 72 | 0.05 | ns |  |  |  |
| 48 vs. 72 | 0.19 | ns |  |  |  |

| Patient 4 | | | | | |
| --- | --- | --- | --- | --- | --- |
| Month 1 aerobic | P Value | Summary | Month 1 anaerobic | P Value | Summary |
| 0 vs. 6 | 0.59 | ns | 0 vs. 6 | 0.26 | ns |
| 0 vs. 12 | 0.05 | ns | 0 vs. 12 | 0.004 | ** |
| 0 vs. 24 | 0.03 | * | 0 vs. 24 | 0.001 | *** |
| 0 vs. 48 | 0.01 | ** | 0 vs. 48 | 0.001 | *** |
| 0 vs. 72 | 0.001 | ** | 0 vs. 72 | 0.01 | ** |
| 6 vs. 12 | 0.05 | * | 6 vs. 12 | 0.01 | * |
| 6 vs. 24 | 0.001 | ** | 6 vs. 24 | 0.002 | ** |
| 6 vs. 48 | 0.004 | ** | 6 vs. 48 | 0.001 | *** |
| 6 vs. 72 | 0.01 | ** | 6 vs. 72 | 0.01 | * |
| 12 vs. 24 | 0.16 | ns | 12 vs. 24 | 0.14 | ns |
| 12 vs. 48 | 0.02 | * | 12 vs. 48 | 0.03 | * |
| 12 vs. 72 | 0.01 | ** | 12 vs. 72 | 0.07 | ns |
| 24 vs. 48 | 0.11 | ns | 24 vs. 48 | 0.20 | ns |
| 24 vs. 72 | 0.02 | * | 24 vs. 72 | 0.19 | ns |
| 48 vs. 72 | 0.04 | * | 48 vs. 72 | 0.95 | ns |
| Month 2 aerobic | P Value | Summary | Month 2 anaerobic | P Value | Summary |
| 0 vs. 6 | 0.27 | ns | 0 vs. 6 | 0.33 | ns |
| 0 vs. 12 | 0.05 | * | 0 vs. 12 | 0.07 | ns |
| 0 vs. 24 | 0.03 | * | 0 vs. 24 | 0.01 | * |
| 0 vs. 48 | 0.02 | * | 0 vs. 48 | 0.01 | ** |
| 0 vs. 72 | 0.01 | * | 0 vs. 72 | 0.004 | ** |
| 6 vs. 12 | 0.57 | ns | 6 vs. 12 | 0.07 | ns |
| 6 vs. 24 | 0.30 | ns | 6 vs. 24 | 0.01 | ** |
| 6 vs. 48 | 0.22 | ns | 6 vs. 48 | 0.003 | ** |
| 6 vs. 72 | 0.10 | ns | 6 vs. 72 | 0.001 | *** |
| 12 vs. 24 | 0.72 | ns | 12 vs. 24 | 0.13 | ns |
| 12 vs. 48 | 0.42 | ns | 12 vs. 48 | 0.05 | ns |
| 12 vs. 72 | 0.07 | ns | 12 vs. 72 | 0.02 | * |
| 24 vs. 48 | 0.97 | ns | 24 vs. 48 | 0.42 | ns |
| 24 vs. 72 | 0.23 | ns | 24 vs. 72 | 0.03 | * |
| 48 vs. 72 | 0.42 | ns | 48 vs. 72 | 0.12 | ns |
| Month 3 aerobic | P Value | Summary |  |  |  |
| 0 vs. 6 | 0.29 | ns |  |  |  |
| 0 vs. 12 | 0.08 | ns |  |  |  |
| 0 vs. 24 | 0.001 | ** |  |  |  |
| 0 vs. 48 | 0.001 | *** |  |  |  |
| 0 vs. 72 | 0.0004 | *** |  |  |  |
| 6 vs. 12 | 0.77 | ns |  |  |  |
| 6 vs. 24 | 0.13 | ns |  |  |  |
| 6 vs. 48 | 0.06 | ns |  |  |  |
| 6 vs. 72 | 0.05 | * |  |  |  |
| 12 vs. 24 | 0.26 | ns |  |  |  |
| 12 vs. 48 | 0.10 | ns |  |  |  |
| 12 vs. 72 | 0.06 | ns |  |  |  |
| 24 vs. 48 | 0.27 | ns |  |  |  |
| 24 vs. 72 | 0.04 | * |  |  |  |
| 48 vs. 72 | 0.51 | ns |  |  |  |

| Patient 5 | | | | | |
| --- | --- | --- | --- | --- | --- |
| Month 1 aerobic | P Value | Summary | Month 2 aerobic | P Value | Summary |
| 0 vs. 6 | 0.35 | ns | 0 vs. 6 | 0.35 | ns |
| 0 vs. 12 | 0.09 | ns | 0 vs. 12 | 0.05 | * |
| 0 vs. 24 | 0.003 | ** | 0 vs. 24 | 0.10 | ns |
| 0 vs. 48 | 0.001 | ** | 0 vs. 48 | 0.002 | ** |
| 0 vs. 72 | 0.0001 | *** | 0 vs. 72 | 0.001 | *** |
| 6 vs. 12 | 0.18 | ns | 6 vs. 12 | 0.40 | ns |
| 6 vs. 24 | 0.08 | ns | 6 vs. 24 | 0.26 | ns |
| 6 vs. 48 | 0.01 | ** | 6 vs. 48 | 0.02 | * |
| 6 vs. 72 | 0.01 | ** | 6 vs. 72 | 0.01 | ** |
| 12 vs. 24 | 0.98 | ns | 12 vs. 24 | 0.84 | ns |
| 12 vs. 48 | 0.27 | ns | 12 vs. 48 | 0.03 | * |
| 12 vs. 72 | 0.11 | ns | 12 vs. 72 | 0.004 | ** |
| 24 vs. 48 | 0.02 | * | 24 vs. 48 | 0.29 | ns |
| 24 vs. 72 | 0.001 | ** | 24 vs. 72 | 0.06 | ns |
| 48 vs. 72 | 0.15 | ns | 48 vs. 72 | 0.02 | * |
| Month 3 aerobic | P Value | Summary |  |  |  |
| 0 vs. 6 | 0.26 | ns |  |  |  |
| 0 vs. 12 | 0.12 | ns |  |  |  |
| 0 vs. 24 | 0.001 | ** |  |  |  |
| 0 vs. 48 | 0.001 | ** |  |  |  |
| 0 vs. 72 | 0.002 | ** |  |  |  |
| 6 vs. 12 | 0.96 | ns |  |  |  |
| 6 vs. 24 | 0.08 | ns |  |  |  |
| 6 vs. 48 | 0.05 | ns |  |  |  |
| 6 vs. 72 | 0.03 | * |  |  |  |
| 12 vs. 24 | 0.09 | ns |  |  |  |
| 12 vs. 48 | 0.05 | ns |  |  |  |
| 12 vs. 72 | 0.03 | * |  |  |  |
| 24 vs. 48 | 0.73 | ns |  |  |  |
| 24 vs. 72 | 0.19 | ns |  |  |  |
| 48 vs. 72 | 0.60 | ns |  |  |  |

| Patient 6 | | | | | |
| --- | --- | --- | --- | --- | --- |
| Month 1 aerobic | P Value | Summary | Month 1 anaerobic | P Value | Summary |
| 0 vs. 6 | 0.00 | *** | 0 vs. 6 | 0.05 | * |
| 0 vs. 12 | <0.0001 | **** | 0 vs. 12 | 0.01 | * |
| 0 vs. 24 | <0.0001 | **** | 0 vs. 24 | 0.003 | ** |
| 0 vs. 48 | 0.001 | ** | 0 vs. 48 | 0.01 | * |
| 0 vs. 72 | 0.003 | ** | 0 vs. 72 | 0.004 | ** |
| 6 vs. 12 | 0.01 | ** | 6 vs. 12 | 0.13 | ns |
| 6 vs. 24 | 0.01 | ** | 6 vs. 24 | 0.03 | * |
| 6 vs. 48 | 0.01 | ** | 6 vs. 48 | 0.03 | * |
| 6 vs. 72 | 0.01 | ** | 6 vs. 72 | 0.01 | * |
| 12 vs. 24 | 0.03 | * | 12 vs. 24 | 0.89 | ns |
| 12 vs. 48 | 0.02 | * | 12 vs. 48 | 0.23 | ns |
| 12 vs. 72 | 0.02 | * | 12 vs. 72 | 0.13 | ns |
| 24 vs. 48 | 0.17 | ns | 24 vs. 48 | 0.12 | ns |
| 24 vs. 72 | 0.07 | ns | 24 vs. 72 | 0.03 | * |
| 48 vs. 72 | 0.19 | ns | 48 vs. 72 | 0.19 | ns |
| Month 2 aerobic | P Value | Summary | Month 3 aerobic | P Value | Summary |
| 0 vs. 6 | 0.01 | * | 0 vs. 6 | 0.08 | ns |
| 0 vs. 12 | 0.01 | ** | 0 vs. 12 | 0.07 | ns |
| 0 vs. 24 | 0.003 | ** | 0 vs. 24 | 0.02 | * |
| 0 vs. 48 | 0.001 | ** | 0 vs. 48 | 0.004 | ** |
| 0 vs. 72 | 0.001 | ** | 0 vs. 72 | 0.003 | ** |
| 6 vs. 12 | 0.27 | ns | 6 vs. 12 | 1.00 | ns |
| 6 vs. 24 | 0.0002 | *** | 6 vs. 24 | 0.27 | ns |
| 6 vs. 48 | 0.02 | * | 6 vs. 48 | 0.01 | ** |
| 6 vs. 72 | 0.02 | * | 6 vs. 72 | 0.004 | ** |
| 12 vs. 24 | 0.12 | ns | 12 vs. 24 | 0.42 | ns |
| 12 vs. 48 | 0.04 | * | 12 vs. 48 | 0.02 | * |
| 12 vs. 72 | 0.04 | * | 12 vs. 72 | 0.01 | ** |
| 24 vs. 48 | 0.53 | ns | 24 vs. 48 | 0.05 | * |
| 24 vs. 72 | 0.53 | ns | 24 vs. 72 | 0.02 | * |
| 48 vs. 72 | >0.9999 | ns | 48 vs. 72 | 0.12 | ns |

| Patient 7 | | | | | |
| --- | --- | --- | --- | --- | --- |
| Month 1 aerobic | P Value | Summary | Month 2 aerobic | P Value | Summary |
| 0 vs. 6 | 0.71 | ns | 0 vs. 6 | 0.005 | ** |
| 0 vs. 12 | 0.005 | ** | 0 vs. 12 | 0.002 | ** |
| 0 vs. 24 | 0.004 | ** | 0 vs. 24 | 0.004 | ** |
| 0 vs. 48 | 0.0004 | *** | 0 vs. 48 | 0.01 | ** |
| 0 vs. 72 | 0.0003 | *** | 0 vs. 72 | 0.001 | *** |
| 6 vs. 12 | 0.15 | ns | 6 vs. 12 | 0.22 | ns |
| 6 vs. 24 | 0.05 | ns | 6 vs. 24 | 0.13 | ns |
| 6 vs. 48 | 0.01 | * | 6 vs. 48 | 0.03 | * |
| 6 vs. 72 | 0.01 | * | 6 vs. 72 | 0.01 | ** |
| 12 vs. 24 | 0.02 | * | 12 vs. 24 | 0.59 | ns |
| 12 vs. 48 | 0.005 | ** | 12 vs. 48 | 0.02 | * |
| 12 vs. 72 | 0.002 | ** | 12 vs. 72 | 0.03 | * |
| 24 vs. 48 | 0.09 | ns | 24 vs. 48 | 0.30 | ns |
| 24 vs. 72 | 0.04 | * | 24 vs. 72 | 0.04 | * |
| 48 vs. 72 | 0.52 | ns | 48 vs. 72 | 0.18 | ns |
| Month 3 aerobic | P Value | Summary |  |  |  |
| 0 vs. 6 | 0.33 | ns |  |  |  |
| 0 vs. 12 | 0.0004 | *** |  |  |  |
| 0 vs. 24 | 0.0003 | *** |  |  |  |
| 0 vs. 48 | 0.003 | ** |  |  |  |
| 0 vs. 72 | 0.0001 | *** |  |  |  |
| 6 vs. 12 | 0.01 | ** |  |  |  |
| 6 vs. 24 | 0.01 | ** |  |  |  |
| 6 vs. 48 | 0.01 | * |  |  |  |
| 6 vs. 72 | 0.002 | ** |  |  |  |
| 12 vs. 24 | 0.42 | ns |  |  |  |
| 12 vs. 48 | 0.05 | ns |  |  |  |
| 12 vs. 72 | 0.005 | ** |  |  |  |
| 24 vs. 48 | 0.13 | ns |  |  |  |
| 24 vs. 72 | 0.01 | * |  |  |  |
| 48 vs. 72 | 0.13 | ns |  |  |  |

**Appendix A.** Significance values for Figure 5.
